# A novel ferroptosis-related genes model for prognosis prediction of lung adenocarcinoma

**DOI:** 10.1186/s12890-021-01588-2

**Published:** 2021-07-13

**Authors:** Fei Li, Dongcen Ge, Shu-lan Sun

**Affiliations:** 1 The First Department of Thoracic Medicine, Cancer Hospital of China Medical University, Liaoning Cancer Hospital and Institute, Shenyang, 110042 Liaoning China; 2 College of Basic Medical Sciences, Dalian Medical University, Dalian, 116044 Liaoning China; 3 Central Laboratory, Cancer Hospital of China Medical University, Liaoning Cancer Hospital and Institute, NO. 44, Xiaoheyan Road, Dadong District, Shenyang, 110042 Liaoning China

**Keywords:** Lung adenocarcinoma, Cox regression analysis, Prognosis prediction model, Ferroptosis

## Abstract

**Background:**

Ferroptosis is a newly discovered form of cell death characterized by iron-dependent lipid peroxidation. This study aims to investigate the potential correlation between ferroptosis and the prognosis of lung adenocarcinoma (LUAD).

**Methods:**

RNA-seq data were collected from the LUAD dataset of The Cancer Genome Atlas (TCGA) database. Based on ferroptosis-related genes, differentially expressed genes (DEGs) between LUAD and paracancerous specimens were identified. The univariate Cox regression analysis was performed to screen key genes associated with the prognosis of LUAD. LUAD patients were divided into the training set and validation set. Then, we screened out key genes and built a prognostic prediction model involving 5 genes using the least absolute shrinkage and selection operator (LASSO) regression with tenfold cross-validation and the multivariate Cox regression analysis. After dividing LUAD patients based on the median level of risk score as cut-off value, the generated prognostic prediction model was validated in the validation set. Moreover, we analyzed the somatic mutations, and estimated the scores of immune infiltration in the high-risk and low-risk groups. Functional enrichment analysis of DEGs was performed as well.

**Results:**

High-risk scores indicated the worse prognosis of LUAD. The maximum area under curve (AUC) of the training set and the validation set in this study was 0.7 and 0.69, respectively. Moreover, we integrated the age, gender, and tumor stage to construct the composite nomogram. The charts indicated that the AUC of LUAD cases with the survival time of 1, 3 and 5 years was 0.698, 0.71 and 0.73, respectively. In addition, the mutation frequency of LUAD patients in the high-risk group was significantly higher than that in the low-risk group. Simultaneously, DEGs were mainly enriched in ferroptosis-related pathways by analyzing the functional results.

**Conclusions:**

This study constructs a novel LUAD prognosis prediction model involving 5 ferroptosis-related genes, which can be used as a promising tool for decision-making of clinical therapeutic strategies of LUAD.

**Supplementary Information:**

The online version contains supplementary material available at 10.1186/s12890-021-01588-2.

## Introduction

The ferroptosis is an iron-dependent form of regulated cell death (RCD) that has been recently discovered. It differs from the apoptosis, necrosis, and autophagy [1]. The implementation of ferroptosis requires the activation of the following three ferroptosis features: The oxidation of phospholipids containing polyunsaturated fatty acids (PUFA); The presence of redox active iron; and the loss of lipid peroxide repair abilities [2]. With the in-depth analysis of ferroptosis, the induction of ferroptosis has been identified as a vital event involved in pathological progressions, including human tumors. Preliminary evidences have suggested the regulatory effect of ferroptosis on the growth of various types of cancers like renal cancer, pancreatic cancer, non-small-cell lung cancer (NSCLC) and diffuse large B-cell lymphoma [3]. The ferroptosis has been identified to suppress tumor growth and the progression, and as a result, the induction of ferroptosis has emerged as a promising anti-cancer treatment [4].

Lung cancer is the most prevalent one among malignancies and it is the chief leading cause of tumor-related deaths worldwide. Pathologically categorized, about 85% of lung cancer cases belong to NSCLC, of which lung adenocarcinoma (LUAD) is one of the most frequent subtypes [5]. With the wide-spreading application of targeted drugs and immune checkpoint inhibitors, therapeutic options of patients with LUAD have revolutionarily changed. However, the prognosis of metastatic or recurrent LUAD is still far away from satisfying [6]. Besides, the overall survival of lung cancer patients significantly varies across the world, with a 5-year survival of 21.2% in the United States, which can be higher than that in China [7]. Recent studies have reported that up-regulation of the GSH synthesis pathway in NSCLC cells can suppress ferroptosis [8],^9^. NFS1, as a ferroptosis-related gene, is detected highly expressed in LUAD cells [10]. In addition, ferroptosis is also correlated to the prognosis of renal carcinoma and hepatocellular carcinoma [11],^12^. We therefore speculated whether ferroptosis is correlated to the prognosis of LUAD, and the possible involvement of ferroptosis-related genes.

RNA sequencing data of ferroptosis-related genes and clinical information of LUAD patients were downloaded from the public databases. It is shown that expression levels of ferroptosis-related genes were correlated to survival outcomes of LUAD. In the present study, LUAD patients were divided into a training set and a validation set based on the random stratified sampling of tumor stages. Then, we established the multi-gene LUAD prognosis prediction model and calculated risk scores through the LASSO regression with tenfold cross-validation, and univariate and multivariate Cox regression analyses. Finally, the established model was verified in the validation set and the overall sample set, aiming to test the fitting degree of the model. To explore the underlying molecular mechanism of the difference in the prognosis of LUAD, we further performed immune and biological functional enrichment analyses.

## Materials and methods

### Data acquisition and preliminary processing

LUAD is a frequently detected subtype of NSCLC. LUAD dataset obtained from the TCGA database (https://portal.gdc.cancer.gov/) involved 533 cancer specimens and 59 paracancerous ones. Their raw RNA-Seq data, single-nucleotide variation (SNV) data and clinical information were included as well. The mRNA expression data were normalized using the DESeq2 variance stabilizing transformation (VST). Based on the previous research, we selected the top 60 ferroptosis-related genes as the candidate gene set (Additional file 5: Table S1) [4, 13–15].

### Analysis of DEGs and model establishment

The DESeq2 package in R [16] was utilized to analyze DEGs between LUAD specimens and paracancerous ones based on the false discovery rate (FDR) < 0.05. The Cox proportional-hazards model is a type of semiparametric regression model, in which survival outcomes and survival time are considered as the dependent variables, and the impact of multiple factors on survival time is analyzed. The most crucial concept in the model is hazard ratio (HR). Generally speaking, HR > 1 and HR < 1 indicate a risk and protective prognostic factor in cancer dataset, respectively. The values in the matrix of VST were utilized to the following univariate Cox regression analysis that assessed potential influences of ferroptosis-related genes on the overall survival of LUAD. *P* values were adjusted with the Benjamini & Hochberg (BH) procedure. Genes with HR ≠ 1 and *p* < *0.05* were selected as prognosis-related genes. Subsequently, the intersection set between the identified DEGs and prognosis-related genes was taken, and their downstream functions in influencing the prognosis of LUAD were further analyzed.

The glmnet package in R was utilized to perform LASSO regression analysis on the target gene set [17] and the establishment of the multi-gene prognostic prediction model. To avoid over-fitting and obtain reliable results, we applied the tenfold cross-validation to acquire optimal lambda values from the minimum partial likelihood deviance, and screened out representative genes. A few candidates were selected to establish a multivariate Cox regression model [18]. Using the coxph function, the PH test of each factor was performed, and the VIF and correlation coefficient of each factor in each regression model were calculated as well. Meanwhile, the possible collinearity of factors was determined. Variables that conformed to the PH hypothesis and collinearity tests were re-modeled. Risk scores of LUAD patients were calculated according to the modeling results. The formula was established as follows: Risk score = Sum (expression level of each gene × corresponding coefficient). Expression level of each gene was the normalized mRNA expression, and the coefficient was the result of the multivariate Cox regression analysis.

To explore the survival predictive feasibility of risk scores obtained from the multi-gene prognostic prediction model, we used the "survival" and "survminer" packages and the Kaplan–Meier method to estimate the survival curve. The time-dependent ROC curve was plotted by the "survival ROC" package in G, which graphically displayed the predictive ability in the prognosis of LUAD at different time points.

### Construction of the prognostic composite nomogram and its verification

To establish a more reliable prediction method that could be applied in clinical practice, we constructed the composite nomogram through the rms package. Similarly, clinical features and risk scores of LUAD patients were combined to establish a multivariate Cox regression model. Based on the influenced degrees of risk factors on the outcome, they were graded to value its level, and a total score was obtained by the sum of the score of each factor. Finally, through the converse relation between the total scores and the outcome events, the predicted survival time of LUAD patients was calculated.

We further verified the accuracy of the nomogram by applying the model using the C-statistic, calibration curve and time-dependent ROC curve. Meanwhile, the nomogram model was calibrated to predict the proximity between the predicted survival and the actual result numerically. The Hosmer–Lemeshow (H–L) test was performed by dividing data into 3 ascending ordered groups (tertiles) based on the predicted result obtained from the model, thus testing the goodness of fit for the χ2 test. Furthermore, the average predicted survival was compared with the actual event rate estimated using the Kaplan–Meier method.

### The enrichment analysis of DEGs function and the enrichment score of immune gene set

DEGs between high-risk groups and low-risk groups divided by risk scores were obtained. Subsequently, enrichment analysis of gene ontology (GO) and Kyoto Encyclopedia of Genes and Genomes (KEGG) pathways of DEGs were analyzed using the clusterprofiler package in R [19].

The single-sample gene set enrichment analysis (ssGSEA) on 29 immune gene sets [20] involving 16 immune cells and 13 immune-related activation pathways was performed using the gsva package in R [21] (Additional file 6: Table S2). The degree of immune infiltration of each sample was recorded.

### Statistical analysis

**Fig. 1:**
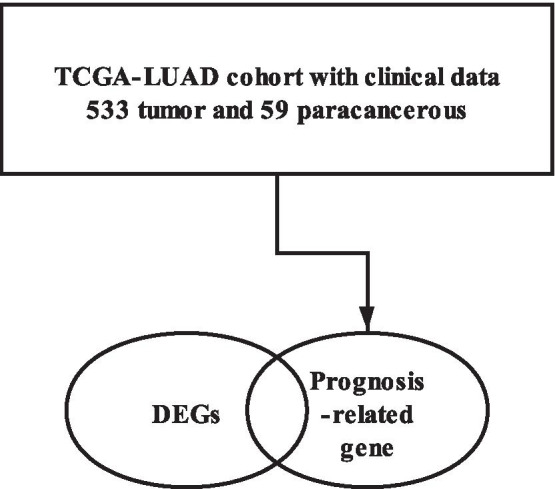
Flow chart of the entire study

The sample function in R was used to perform the random stratified sampling. Meanwhile, the heatmap of DEGs and SNV mutation landscape were respectively depicted by the pheatmap and maftools packages in R [22]. Wilcoxon-test was used to test the difference between groups of the boxplot. Kaplan–Meier survival curve was depicted for assessing the prognostic potential, followed by the log-rank test to compare the difference between curves. The R software (Version 3.6.0) was used for all statistical analyses. The flowchart of this study was shown in Fig. 1.

## Results

### Identifying prognosis- related DEGs that were associated with ferroptosis

**Fig. 2:**
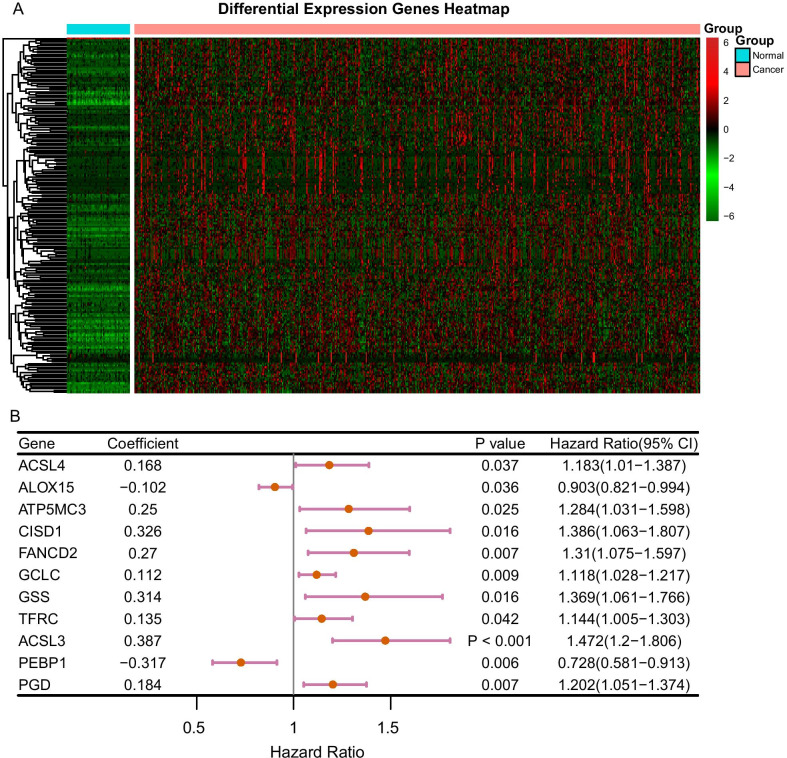
Data processing, and screening of the DEGs. **a** Heatmap of the top 200 DEGs in LUAD. **b** Univariate Cox regression analysis of 11 prognostic genes from 60 ferroptosis genes

The downloaded LUAD dataset contained 533 LUAD specimens and 59 paracancerous ones. Among them, 502 LUAD cases had clinical information of the overall survival (Table 1). Besides, the DESeq2 package was used to analyze the mRNA expression data of the original counts for obtaining DEGs (FDR < 0.05). The acquired DEGs were sorted according to the value of log2 fold change, and they were displayed by depicting the heatmap (Fig. 2a). Univariate Cox regression analysis revealed that among the 60 ferroptosis-related genes, 11 were significantly correlated to the prognosis of LUAD (*adj p* < 0.05). Taking the intersection of the 11 genes and DEGs, it is obviously shown that the former ones were differentially expressed between LUAD and paracancerous specimens. In addition, the results of Cox regression analysis were depicted in the forest plots (Fig. 2b).

**Table 1:** Clinical characteristics of LUAD patients

Clinical characteristics of LUAD patients		
	Training set	Validate set
No. of patient	251	251
Age (mean (SD))	64.57 (10.05)	65.86 (10.04)
Gender (%)		
Male	123 (49.0)	109 (43.4)
Female	128 (51.0)	142 (56.6)
Stage (%)		
Stage I	1 (0.4)	4 (1.6)
Stage IA	73 (29.4)	57 (23.2)
Stage IB	63 (25.4)	72 (29.3)
Stage II	1 (0.4)	0 (0.0)
Stage IIA	22 ( 8.9)	28 (11.4)
Stage IIB	37 (14.9)	31 (12.6)
Stage IIIA	35 (14.1)	35 (14.2)
Stage IIIB	3 ( 1.2)	7 (2.8)
Stage IV	13 ( 5.2)	12 (4.9)
Pathologic T (%)		
T1	38 (15.1)	27 (10.8)
T1a	29 (11.6)	19 (7.6)
T1b	24 (9.6)	31 (12.4)
T2	78 (31.1)	82 (32.7)
T2a	40 (15.9)	41 (16.3)
T2b	11 (4.4)	16 (6.4)
T3	23 (9.2)	22 (8.8)
T4	7 (2.8)	11 (4.4)
TX	1 (0.4)	2 (0.8)
Pathologic N (%)		
N0	164 (65.3)	162 (64.8)
N1	47 (18.7)	47 (18.8)
N2	32 (12.7)	37 (14.8)
N3	1 (0.4)	1 (0.4)
NX	7 (2.8)	3 (1.2)
Pathologic M (%)		
M0	160 (64.3)	174 69.9)
M1	7 (2.8)	10 (4.0)
M1a	1 (0.4)	1 (0.4)
M1b	4 (1.6)	1 (0.4)
MX	77 (30.9)	63 (25.3)
Vital status = Dead (%)	92 (36.7)	90 (35.9)
Race (%)		
American indian or alaska native	1 (0.4)	0 (0.0)
Asian	4 (1.6)	3 (1.2)
Black or african american	26 (10.4)	26 (10.4)
Not reported	26 (10.4)	28 (11.2)
White	194 (77.3)	194 (77.3)
Smoke_history = Yes (%)	88 (35.1)	99 (39.4)
OS time (mean)	890.17	926.26

### The multi-gene prognostic model in the training set

**Fig. 3:**
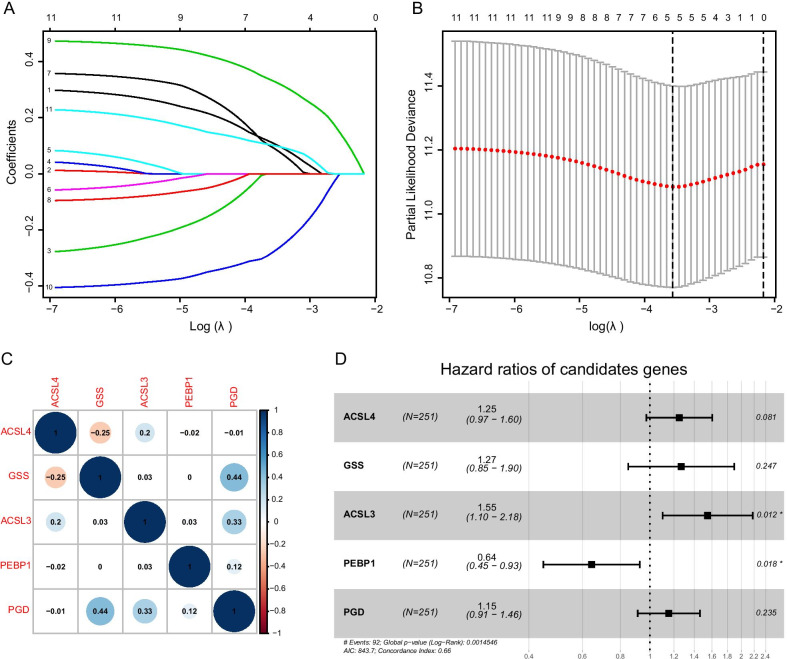
Constructing five-gene-based classifier by LASSO Cox regression model. **a** Trajectory of each independent variable. Horizontal axis represents log of independent variable λ. Vertical axis represents coefficient of independent variable. **b** tenfold cross-validation of tuning parameters in LASSO model. **c** Correlation between candidate genes. **d** Forest plot for five-gene signature screened by LASSO Cox regression

**Fig. 4:**
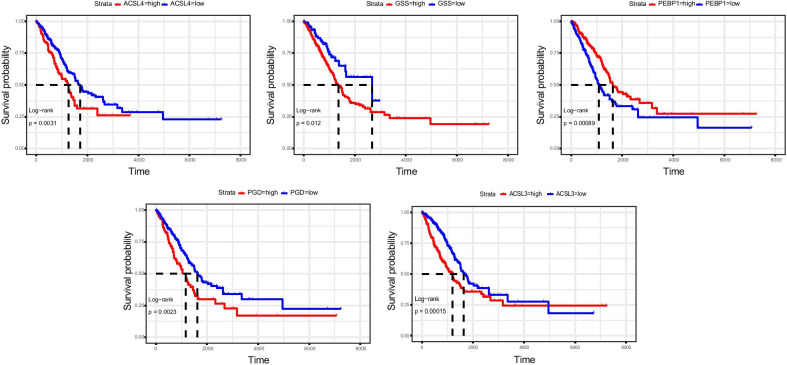
Survival analysis of the five candidate genes. Survival analysis of five candidate genes using the best out‐based cut‐point calculated by the surv_cutpoint function. High expressions of four genes indicate a worse prognosis of LUAD. A lower expression of PEBP1 indicates a better prognosis of LUAD. A log-rank test was used to measure statistical significance

**Fig. 5:**
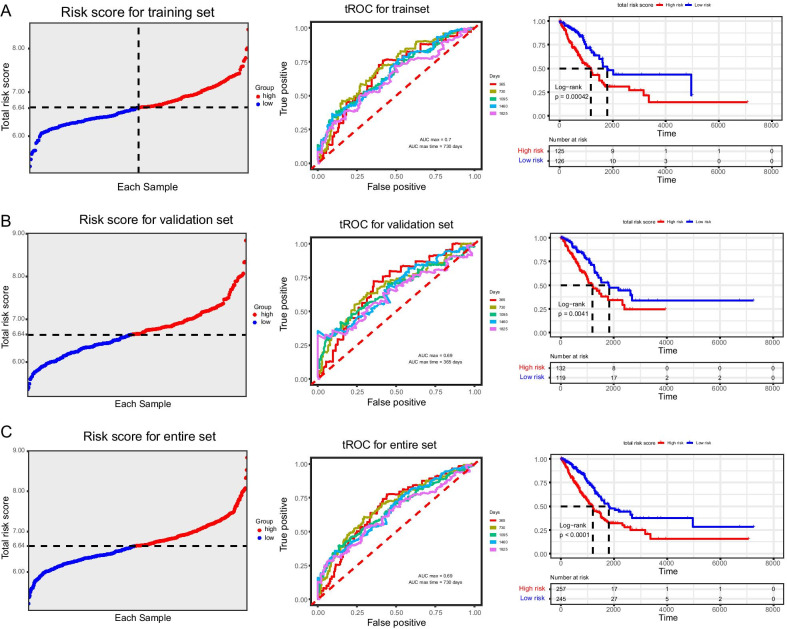
Prognostic analysis of five-gene signature. Prognostic analysis of five-gene signature in **a** the training set, **b** the internal validation set and **c** the entire set. The dotted line represents the median risk score and divided the patients into low-risk group and high-risk group. The curve of risk score. Time-dependent ROC analysis the of the five-gene signature. ROC receiver operating characteristic. Kaplan–Meier survival analysis of the five-gene signature

We randomly stratified LUAD patients according to their tumor stage and divided them into the training set (n = 251) and validation set (n = 251). Meanwhile, LASSO-Cox regression analysis of the above-mentioned set was performed to identify prognosis-related genes that were correlated to ferroptosis (Fig. 3a, b). The results revealed that a total of 5 genes conformed to the pH-partition hypothesis (Additional file 1: Figure S1) and the collinearity test (correlation coefficient less than 0.5) (Fig. 3c), namely the *ACSL4*, *GSS*, *ACSL3*, *PEBP1* and *PGD* genes. Through plotting the forest plots of them, it is disclosed that the *ACSL4* (*adj P* < 0.05), *GSS*, *ACSL3* and *PGD* genes were the risk factors for the prognosis of LUAD, while the *PEBP1* gene was a protective factor (*adj P* < 0.05) (Fig. 3d). Furthermore, LUAD patients were subgrouped based on the expression level of the 5 identified genes, and the corresponding Kaplan–Meier survival curves were displayed in Fig. 4. Besides, the threshold of the expression level of each gene was calculated by the surv_cutpoint function from the survminer R package (Additional file 2: Figure S2). We subsequently calculated the risk score of each sample based on the modeling results using the following formula: Risk score = 0.2226 × expression level of *ACSL4* + 0.2373 × expression level of *GSS* + 0.4369 × expression level of *ACSL3*—0.4417 × expression level of *PEBP1* + 0.1431 × expression level of *PGD*. Expression level of each gene was normalized. LUAD patients were further divided into the high-risk group and low-risk group based on the individualized risk score. The grouped threshold was determined by the median value of the risk score of all LUAD patients in the training set, the median of which was 6.64. Kaplan–Meier survival curves based on the overall survival calculated using the KM algorithm have indicated that LUAD patients in the high-risk group had a worse survival than that of the low-risk group (*p* = 0.0041). Later, the predictive potential of the risk score model in LUAD was assessed by depicting the ROC curve, and the highest AUC was 0.7 (Fig. 5a).

### Verification of the prognostic model

To determine the reliability of the established model and the predictive accuracy, results obtained from the prognostic model were validated in the verification set. Similarly, risk scores in the validation set, and subgroup classification of LUAD patients into the high-risk group and low-risk group with the same threshold (cut-off = 6.64) were conducted. Moreover, the survival status of LUAD patients in the validation set was estimated using the KM algorithm, followed by plotting the survival curve. It is consistently shown that the prognosis of LUAD patients in the high-risk group was significantly worse than that of the low-risk group (*p* = 0.00042), and the maximum AUC was 0.69 (Fig. 5b). Finally, we combined the training and validation set, calculated the risk score of the 502 patients according to the similar models, and performed survival analysis and plotted the survival curves of the high-risk group (n = 257) and low-risk group (n = 245) using the same threshold (Fig. 5c). As expected, LUAD patients in the high-risk group presented a worse prognosis (*p* < 0.0001), and the maximum AUC was 0.69.

### Construction and verification of the composite nomogram combining clinical information

**Fig. 6:**
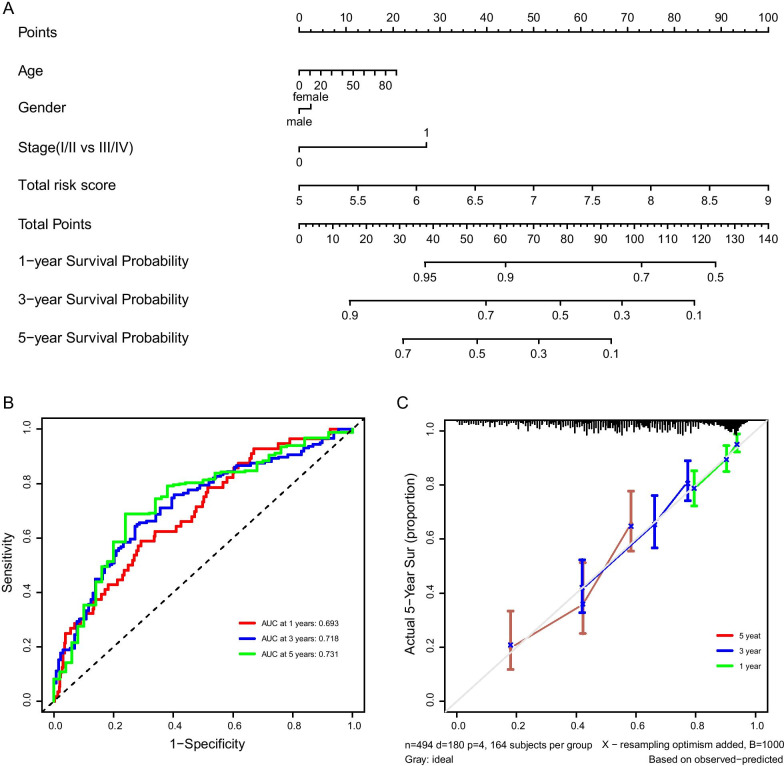
Construction of nomogram prognostic model. **a** Nomogram integrated 5-genes-based risk score and the significant clinical traits. **b** The time-dependent ROC curves of the nomogram and the clinical traits in the entire LUAD cohort. **c** Calibration plot of actual risk probability

To better apply the model to the actual clinical situation, clinical features of LUAD patients were introduced into the evaluations of the model and thus a composite nomogram to predict the survival probabilities of LUAD patients was constructed. By introducing the age, gender, tumor stage (I/II vs III/IV) and risk score as variables in the multivariate Cox regression analysis, and incorporating the above factors into the model as variables, a nomogram was obtained (Fig. 6a), in which, the line segment corresponding to each variable was marked with a scale that represented the value range of the variable, and the length of the line segment reflected the contribution of the factor to survival. The points in the nomogram represented the individual scores corresponding to each variable under different values and total points. The corresponding individual scores after introducing all the variables were summed to produce the total score. The last three lines represented the total score of 1-year, 3-year and 5-year survival rate. To verify the prognostic value of the nomogram model, the predictive ability of the nomogram was evaluated by tROC, and the AUC corresponding to 1-year, 3-year and 5-year survival was 0.698, 0.718 and 0.731, respectively (Fig. 6b). The prediction result of the C statistics on the nomogram model was 0.677 (95%CI, 0.633–0.721). The check chart indicated a good agreement between the predicted and actual results (Fig. 6c).

### SNV mutation landscape variation between the high-risk group and low-risk group

**Fig.7:**
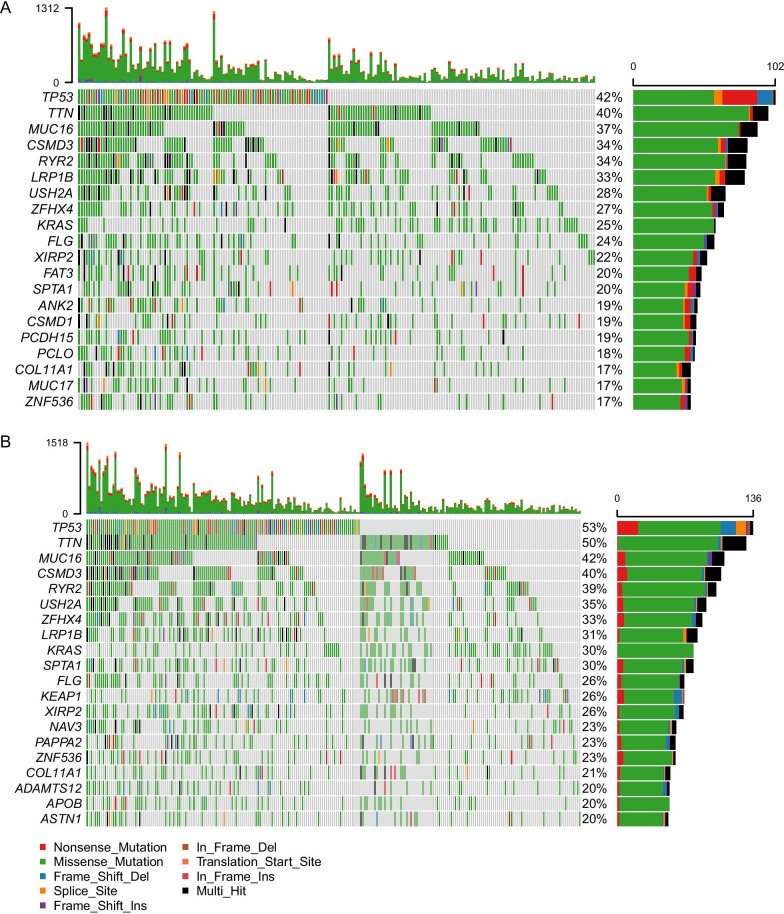
SNV mutation landscape variation between high and low risk groups. Oncoplot displaying the somatic landscape of LUAD with **a** the low-risk group and **b** high-risk group, the genes are sorted according to their mutation frequency

We downloaded the SNV data of 502 LUAD patients, and subgrouped based on the risk score. Each figure of the mutation landscape indicated the top 20 genes with the highest mutation frequency, and their mutations types were labeled with different colors (Fig. 7). The upper and right bar graph represented the total number of mutations in each sample, and the mutation frequency of each gene in all samples. It is revealed that genes with a higher mutation frequency in the high-risk group were consistent with those in the low-risk group, while their mutation frequencies were higher in the high-risk group [*TP53* (53%), *TTN* (50%), *MUC16* (42%), *CSMD3* (40%), and *RYR2* (39%)].

### The GO and KEGG enrichment analysis

**Fig.8:**
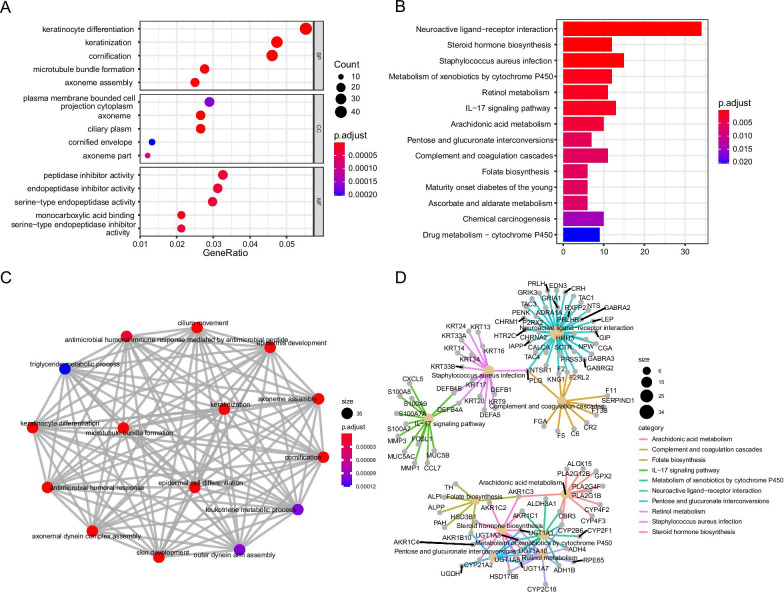
Functional enrichment analysis of DEGs between high risk and low risk group. The more genes enriched in the terms, the darker the color. **a** The top 5 GO terms of each sub-class. **b** The 14 KEGG pathway of key genes. **c** The gene overlap relationship between the enriched GO terms. Overlapped differentially expressed genes of two GO terms indicate the two nodes have an overlap relationship, which are connected by lines in the graph. **d** The relationship between gene and enriched KEGG pathways is demonstrated. If a gene is located under a KEGG pathways, the gene is believed to connect with the KEGG pathway

To further explore the differences in biological functions and pathways between the high-risk group and low-risk group, the DESeq2 package was used to analyze expression levels of DEGs between the high-risk group and low-risk group (logFC > 1, FDR < 0.05). DEGs screened out from both groups were later subjected to GO and KEGG enrichment analysis. The results of pathway enrichment and GO enrichment were shown in Fig. 8, and Additional file 7, 8: Table S3–S4. It is shown multiple pathways were enriched in the metabolism. In detail, the mainly enriched pathways included the neuroactive ligand-receptor interaction, metabolism of xenobiotics by cytochrome P450, steroid hormone biosynthesis, staphylococcus aureus infection pathway, IL-17 signaling pathway, retinol metabolic pathway, etc. The results of GO enrichment showed that the biological functions and processes of DEGs were mainly enriched in the keratinization, axoneme assembly, and microtubule bundle formation. Enrichment results could support the reasons for the variations in the prognosis of LUAD from a functional level. Moreover, some pathways and functions were identified closely correlated to ferroptosis or iron metabolism, which provided references for further research.

### The correlation between risk score and immune status in LUAD

**Fig. 9:**
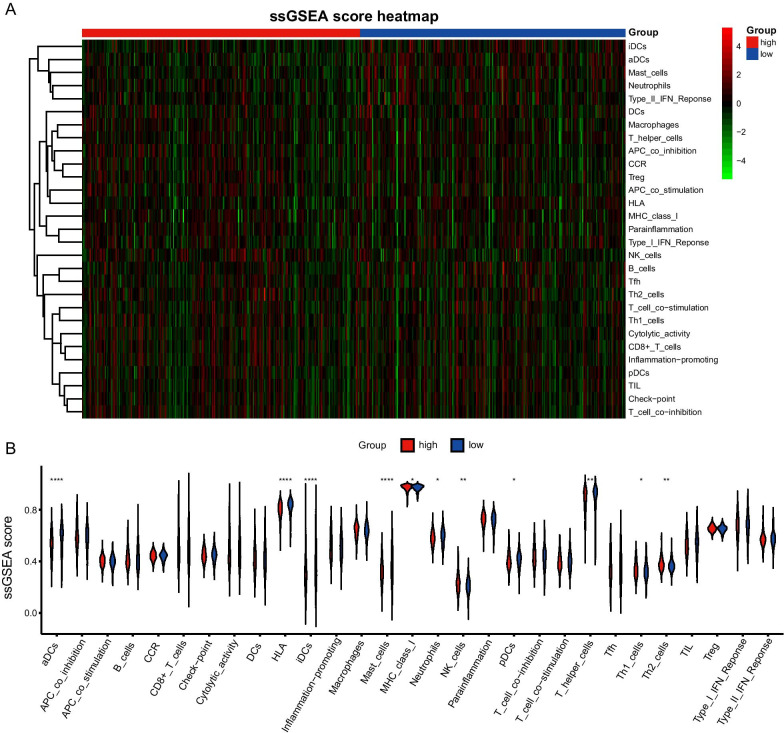
The correlation between risk score and immune status. **a** Heatmap for ssGSEA score in 29 immune genes set of each sample. **b** Differential ssGSEA score between the high-risk group and low-risk group

According to the threshold of risk score, the correlation between risk score and immune status in LUAD patients was further explored. We obtained a total of 29 immune-related gene sets involving multiple immune cells and immune-related functions or pathways. To quantify the enrichment degree of transcriptome data in the 29 immune gene sets, separate enrichment scores for each paring of a sample between the high-risk risk and low-risk group were estimated by the ssGSEA and compared by depicting a boxplot (Fig. 9). The enrichment scores of concentrations of immune cell genes in aDCs, iDCs, B-cells, mast cells, neutrophils, NK cells, T helper cells, Th2 cells and TIL were significantly different between the high-risk group and low-risk group (*p* < 0.05). Besides, the enrichment scores of these immune function gene sets in HLA, inflammation promoting, MHC class I, T cell co-stimulation and type II IFN response were also significantly different between groups (*p* < 0.05).

## Discussion

The current study systematically identified the correlation between 60 ferroptosis-related genes and the prognosis (overall survival) of LUAD. In this study, the LUAD dataset was divided into the training set and validation set by the random stratified sampling method. The prognostic prediction model involving 5 genes was established for the training set samples through the LASSO regression with tenfold cross-validation and univariate and multivariate Cox regression analysis, which was validated in the actual clinical practice.

Previous evidences have confirmed the vital functions of ferroptosis-related genes in the entire ferroptosis process. Nevertheless, the specific influence of a single ferroptosis-related gene on the prognosis of a certain type of cancer remains unclear. We combined the mRNA expression levels of each gene with actual clinical characteristics to analyze the differential expression between LUAD and paracancerous specimens. Moreover, quantitative data of gene expressions were analyzed for their predictive potential in the survival of LUAD. Subgrouped by the risk score, identified genes between the high-risk group and low-risk group were analyzed for their differential variations, biological functions and pathways they were mainly enriched in. In conclusion, our screened DEGs in LUAD patients may be potential targets involved in the occurrence and development of LUAD.

In the constructed predictive model, a total of 5 genes (*ACSL3*, *ACSL4*, *GSS*, *PEBP1*, *PGD*) were involved in, which were closely associated with the process of ferroptosis. The *ACSL3* gene is responsible for exogenous monounsaturated fatty acids to protect cells against ferroptosis, and it is negatively correlated with ferroptosis sensitivity [23]. The *ACSL4* gene is essential for proferroptosis. Knockdown of ACSL4 inhibits erastin-induced ferroptosis, and its overexpression can restore ferroptosis sensitization. Re-expression of flag-tagged human wild-type (WT) ACSL4 (ACSL4-Flag) in Acsl4 KO (Acsl4^−/−^) Pfa1 cells restores full sensitivity to ferroptosis induction, and knockdown of it significantly prolongs survival compared to vehicle-treated mice. Knockout of ACSL4 in ferroptosis-sensitive cells protects erastin- and RSL3-induced cell death [24], 25. The *GSS* gene provides instructions for making glutathione synthetase. The glutathione-dependent lipid hydroperoxidase GPX4 contributes to prevent ferroptosis by converting lipid hydroperoxides into non-toxic lipid alcohols. Overexpression of PEBP1 increases the sensitivity of HK2 cells to RSL3, and knockdown of PEBP1 in HAEC and HT22 cells yields an opposite result [26]. The *PGD* gene is involved in erastin-induced ferroptosis [1].We modeled and calculated the corresponding risk score based on the expression data of 5 candidate genes, and then divided LUAD patients into high-risk group and low-risk group. Our established model effectively predicted the survival of LUAD patients in the training set, the validation set and the total cases. Meanwhile, the gender, age, and tumor stage of LUAD patients were taken into consideration, and thus a composite nomogram was established, which was much closer to the actual clinical practice. Among them, the gender and age of LUAD patients had relatively a small effect on the prognosis, whereas the tumor staging and risk score posed a greater one. The above results were consistent with our investigation expectations.

To explore the factors for the prognosis difference between the high-risk group and low-risk group, we compared the SNV levels and analyzed the differences in biological functions of the two groups. After subgrouping the acquired SNV statistics according to high-risk and low-risk scores, the mutation frequency of each gene and the number of mutations in each sample were calculated, which were displayed as the mutation landscape. The results revealed that LUAD patients in the high-risk group presented a higher frequency of each gene mutation. Moreover, the top 5 genes with the highest mutation frequency in the high-risk group, involving the *TP53* (53%), *TTN* (50%), *MUC16* (42%), *CSMD3* (40%) and *RYR2* (39%) genes presented 5–13% higher mutation frequency than those in the low-risk group. Calculating the mutation frequency easily identified the source of the variations between groups. Genomic analyses about the prognosis difference between high-risk and low-risk LUAD patients need to be performed in the future.

Functional enrichment analysis and immune infiltration score on DEGs between the two groups were further performed. DEGs were mainly enriched in the cytochrome P450, steroid hormones, IL-17 signaling pathways, staphylococcus aureus infection pathways, etc. The cytochrome P450 oxidoreductase (POR) is necessary for triggering the ferroptosis in cancer cells [27]. Moreover, POR boosts the execution of ferroptosis by engaging in the peroxidative modification of phospholipids in the cell membrane. PPARα, as a member of the steroid hormone receptor superfamily, can inhibit iron overload and lead to ferroptosis by combining with the GPX4 and facilitating its expression. The preliminary evaluations indicated that the steroid hormones in adrenal sebaceous cells are significantly affected by ferroptosis induction [28]. Iron plays a crucial role in the continuous evolution process for *staphylococcus aureus* by establishing efficient iron transportation systems. On the one hand, *staphylococcus aureus* consumes hemoglobin in the red blood cells of the host and serum through the transport systems encoded by the iron-regulated surface determinants located in cell wall. On the other hand, it acquires the iron by the siderophores with a high affinity for iron. A previous study has shown that the reactive oxygen species can result in the drug resistance in *staphylococcus aureus* infection [29]. Previous GO enrichment revealed that biological functions like microtubule bundle formation and keratinization are mainly enriched in the iron [30, 31]. Therefore, we concluded that the variations in these pathways might be the inherent factors for the differences between the high-risk group and low-risk group. Our findings provide theoretical references for underlying the mechanism of ferroptosis in lung cancer patients.

Although constructing a prognostic model is of great significance in the TCGA-LUAD cohort, only internal verification and overall verification were performed in the verifying process of the model. Specifically, the external validation set was not included in the examination of the final results. In functional analysis, enrichment analysis was performed in DEGs between the high-risk group and low-risk group, while functions of these pathways were not be experimentally verified. As a result, we only identified which pathways and functions were responsible for triggering the prognosis difference in high-risk and low-risk LUAD patients, but how they induced it needs to be further explored.

In general, we established a prognostic prediction model based on 5 ferroptosis-related genes by analyzing the LUAD dataset. In the training and validation set, this model was found effectively predict the overall survival of LUAD, and more important, clinical features of LUAD patients were taken into consideration, which remarkably simulated the actual clinical practice. Our findings provided a promising tool in predicting the prognosis of LUAD patients, and theoretical references for explaining the prognosis difference between high-risk and low-risk LUAD.

## Conclusions

This study constructed a novel LUAD prognosis prediction model based on 5 ferroptosis-related genes, which can provide a reliable prognostic evaluation tool for clinical practice and assist the clinical therapeutic decision.

## Supplementary Information

**Additional file 1:** **Supplementary figure 1**. COX regression PH hypothesis test for each candidate.

**Additional file 2: Supplementary figure 2**. Gene expression threshold setting.

**Additional file 3.** Supplementary figure legends

**Additional file 4.** Supplementary table legends

**Additional file 5: Supplementary table 1**. Ferroptosis-related genes set

**Additional file 6: Supplementary table 2**. Gene list of immune cells and immune-related functions.

**Additional file 7: Supplementary table 3**. GO enrichment results of DEGs between high risk group and low risk group.

**Additional file 8: Supplementary table 4**. Pathway enrichment results of DEGs between high risk group and low risk group.

## Data Availability

All data generated or analyzed during this study are included in this published article.
