# Supplementary material for: A novel ferroptosis-related genes model for prognosis prediction of lung adenocarcinoma
Source: BMC Pulm Med. 2021 Jul 13;21:229. doi: 10.1186/s12890-021-01588-2 (PMC8276441; doi:10.1186/s12890-021-01588-2)
Supplement: Supplementary file 1 — Additional file 1: Supplementary figure 1. COX regression PH hypothesis test for each candidate. [file 12890_2021_1588_MOESM1_ESM.pdf]

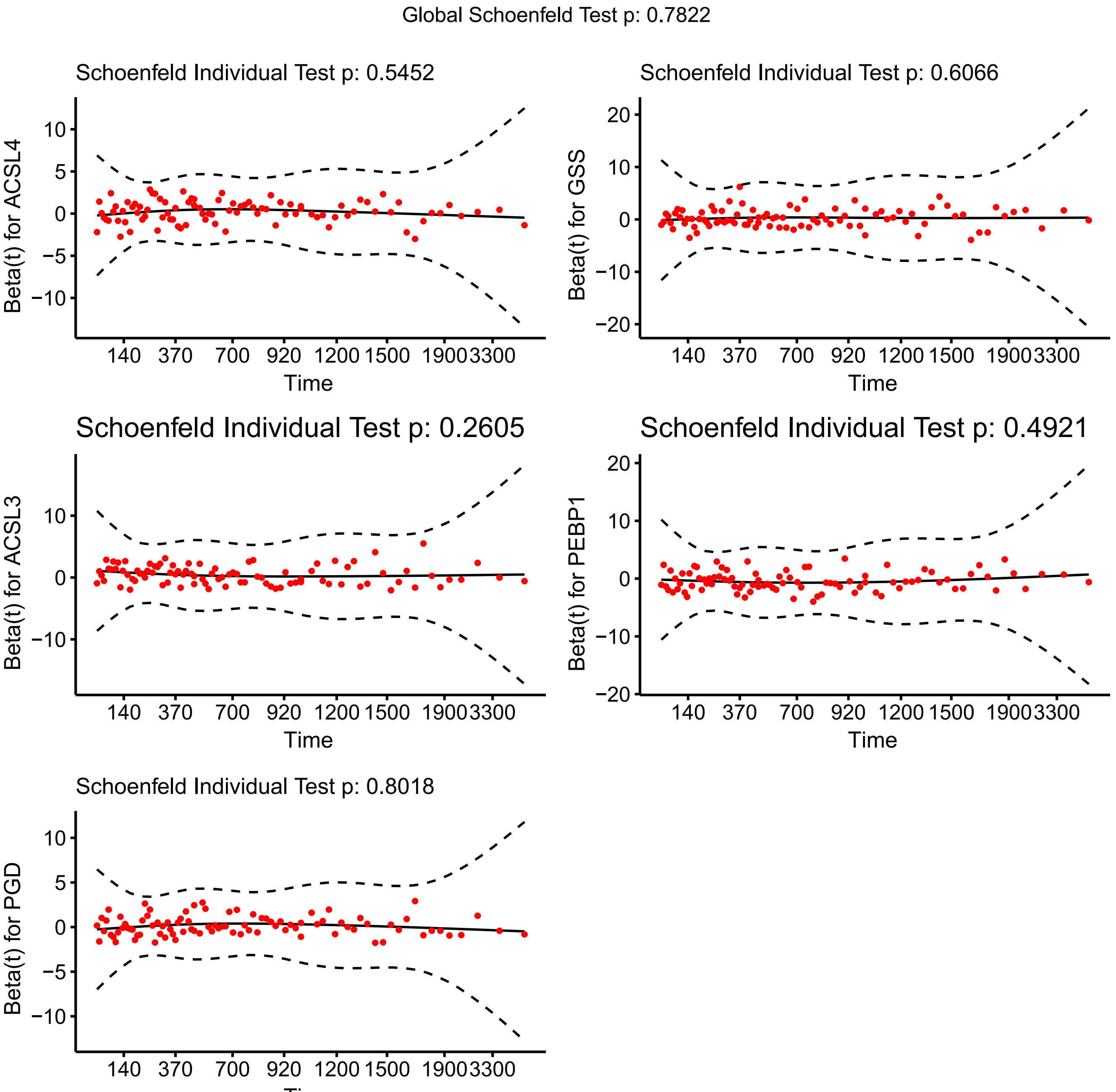

sFigure 1 COX regression PH hypothesis test for each candidate.
