# Supplementary figures and images for: A novel ferroptosis-related genes model for prognosis prediction of lung adenocarcinoma
Source: BMC Pulm Med. 2021 Jul 13;21:229. doi: 10.1186/s12890-021-01588-2 (PMC8276441; doi:10.1186/s12890-021-01588-2)

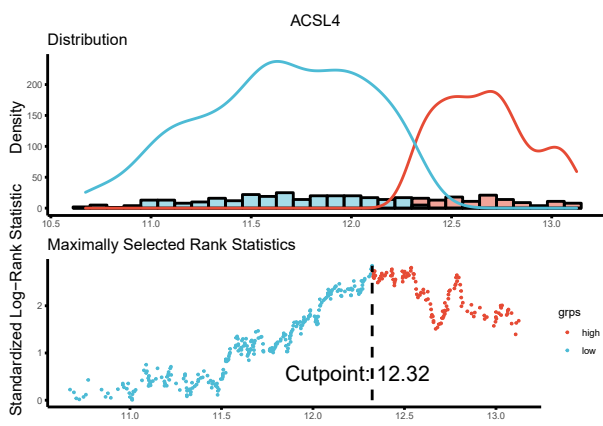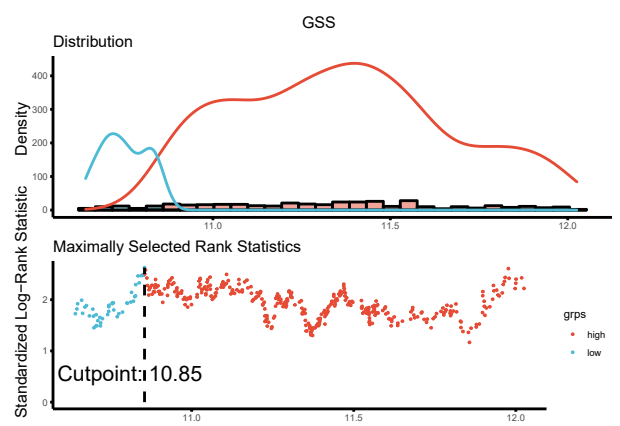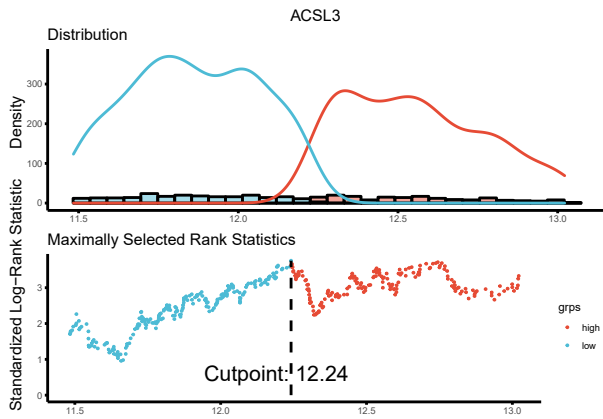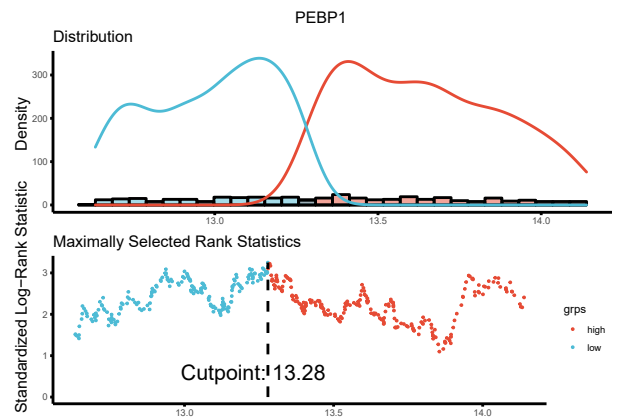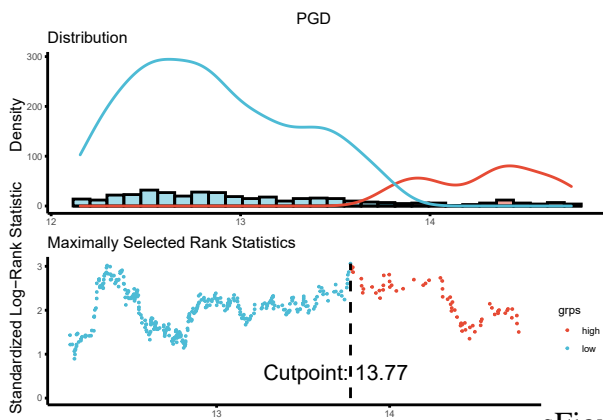

Figure 2 Gene expression threshold setting.

Supplement: Supplementary file 2 — Additional file 2: Supplementary figure 2. Gene expression threshold setting. [file 12890_2021_1588_MOESM2_ESM.pdf]
