# Supplementary material for: A novel ferroptosis-related genes model for prognosis prediction of lung adenocarcinoma
Source: BMC Pulm Med. 2021 Jul 13;21:229. doi: 10.1186/s12890-021-01588-2 (PMC8276441; doi:10.1186/s12890-021-01588-2)
Supplement: Supplementary file 3 — Additional file 3. Supplementary figure legends [file 12890_2021_1588_MOESM3_ESM.doc]

**Fig. s1. COX regression PH hypothesis test for each candidate.** The P value of the five variables is greater than 0.05, indicating that each candidate meets the PH test, while the overall test P value of the model is 0.7822, and the model meets the PH test.

**Fig. s2. Gene expression threshold setting.Statistics the expression distribution of each gene**. Use the surv_cutpoint function to calculate the optimal cut point for each gene.
