# Supplementary material for: A novel ferroptosis-related genes model for prognosis prediction of lung adenocarcinoma
Source: BMC Pulm Med. 2021 Jul 13;21:229. doi: 10.1186/s12890-021-01588-2 (PMC8276441; doi:10.1186/s12890-021-01588-2)
Supplement: Supplementary file 4 — Additional file 4. Supplementary table legends [file 12890_2021_1588_MOESM4_ESM.doc]

**Table s1. ferroptosis-related genes set**

**Table s2. Gene list of immune cells and immune-related functions.**

**Table s3. Pathway enrichment results of DEGs between high risk group and low risk group.**

**Table s4. GO enrichment results of DEGs between high risk group and low risk group.**
